# Epigenetic Conservation Is a Beacon of Function: An Analysis Using Methcon5 Software for Studying Gene Methylation

**DOI:** 10.1200/CCI.19.00109

**Published:** 2020-02-20

**Authors:** Emil Hvitfeldt, Chao Xia, Kimberly D. Siegmund, Darryl Shibata, Paul Marjoram

**Affiliations:** ^1^ Biostatistics Division, Department of Preventive Medicine, Keck School of Medicine, University of Southern California, Los Angeles, CA; ^2^ Department of Pathology, Keck School of Medicine, University of Southern California, Los Angeles, CA

## Abstract

**PURPOSE:**

Different epigenetic configurations allow one genome to develop into multiple cell types. Although the rules governing what epigenetic features confer gene expression are increasingly being understood, much remains uncertain. Here, we used a novel software package, Methcon5, to explore whether the principle of biologic conservation can be used to identify expressed genes. The hypothesis is that epigenetic configurations of important expressed genes will be conserved within a tissue.

**MATERIALS AND METHODS:**

We compared the DNA methylation of approximately 850,000 CpG sites between multiple clonal crypts or glands of human colon, small intestine, and endometrium. We performed this analysis using the new software package, Methcon5, which enables detection of regions of high (or low) conservation.

**RESULTS:**

We showed that DNA methylation is preferentially conserved at gene-associated CpG sites, particularly in gene promoters (eg, near the transcription start site) or the first exon. Furthermore, higher conservation correlated well with gene expression levels and performed better than promoter DNA methylation levels. Most conserved genes are in canonical housekeeping pathways.

**CONCLUSION:**

This study introduces the new software package, Methcon5. In this example application, we showed that epigenetic conservation provides an alternative method for identifying functional genomic regions in human tissues.

## INTRODUCTION

It would be valuable to identify or rank the genes that are critical to the function of a given cell type or tissue. In theory, the epigenetic configuration of a gene should indicate its function, because epigenetics allows many different cell types to develop from a single genome by differentially marking specific genes for expression or silencing.^1^ However, the relative functional importance of the epigenetic elements that cover genomes is a controversial issue, because a relatively large proportion of the human genome can be annotated with different biochemical assays.^2^ One approach to inferring whether a genomic region is functional is to use evolutionary information: functional elements are conserved or constrained between species because of negative or purifying selection.^3^ The idea is that regions with biologic function are under selection and will change at rates lower than regions without function. For example, exonic regions are more conserved than intronic regions. Epigenetic marks are found in both conserved and nonconserved genomic regions.^2^

**CONTEXT**

**Key Objective**
We assessed whether conservation of methylation sites can be used as a proxy for gene expression (ie, do highly conserved CpGs sites within a gene tend to indicate higher expression of that gene?). The relative functional importance of the epigenetic elements that cover genomes is a controversial issue. This study adds to our knowledge of this issue.
**Knowledge Generated**
We demonstrated that genes that are more conserved in terms of their methylation status do tend to be more highly expressed. Furthermore, we demonstrated that, contrary to some prior speculation, conservation of promoter regions does not correlate with gene expression in this way.
**Relevance**
Although this study focuses on three tissue types, we provide a software package to allow other users to easily conduct a similar analysis for data of interest (eg, other tissue types).

In this paper, we provide a software tool, Methcon5, that allows the user to explore whether epigenetic conservation between similar cells in the same and different individuals can reveal biologic function. Epigenetic marks occupy a large proportion of the human genome,^2^ and it is uncertain whether they are all equally functional. Similar to sequence conservation between species,^1^ the idea is that, if an epigenetic configuration is important in a given cell type, it will be the same between cells, because any changes will decrease cell fitness and be subject to negative selection. By contrast, if an epigenetic configuration is unimportant to the cell, its pattern may drift and therefore become different between cells.

Although the method we used is broadly applicable, we here used DNA methylation of CpG sites to test the hypothesis. DNA methylation patterns show somatic inheritance and usually are copied between cell divisions, but the replication fidelity of DNA methylation is relatively low, and changes (methylation to demethylation, or de novo methylation) commonly can be observed within a human lifespan.^4^ Furthermore, DNA methylation can be measured with high reproducibility using the Illumina MethylationEPIC BeadChip Infinium microarrays (Illumina, San Diego, CA),^5^ reducing the experimental background that can confound measures of similarity. We examined the methylation at approximately 850,000 CpG sites in 32 crypts/glands from the human colon, small intestine, and endometrium from eight different individuals. We present novel software and algorithms that measured epigenetic conservation and identified and ranked genes that were preferentially conserved in these human tissues. Consistent with the hypothesis that DNA methylation important to the function of a cell shows epigenetic conservation, we found that the methylation of CpG sites in genes, promoters, housekeeping genes, and more highly expressed genes are, on average, more conserved.

## MATERIALS AND METHODS

### Data

The data consisted of 32 samples of normal tissue taken from the colon, small intestine, and endometrium of eight human participants. Each sample consisted of a pool of approximately 500-10,000 cells from individual crypts or glands. We then assayed those samples using the Illumina MethylationEPIC BeadChip Infinium microarray, which measures methylation at approximately 850,000 CpG sites using hybridization-ligation.^5^ It can measure DNA from paraffin-embedded tissues, single tumor glands (approximately 100 ng), and it has high technical reproducibility (replicates with a Pearson correlation of 0.997^5^). In this example study, we measured the proportion of cells that were methylated at each assayed CpG position for each sample. We then contrasted the results with measurements of gene expression taken from the Expression Atlas, a European Bioinformatics Institute resource that provides gene expression results from > 3,000 experiments from 40 different organisms.^6^

### Statistical Approach

The analysis tool, Methcon5, implemented using the statistical programming language R, version 3.6.1, employs statistical methods to enabled us to assess how DNA methylation varies along the genome, and in specific regions/genes, with a goal of identifying genes with particularly highly conserved DNA methylation.^7^ We used a bootstrapping approach for this problem. We then assessed whether such conserved regions were indicative of genes that are highly expressed in the tissue of interest.

Specifically, within each gene of interest, for every pair of samples, we calculated the Manhattan distance^8^ between all CpG sites at which we had measurements of methylation proportion for that gene. We then calculated the mean of those values across all pairs of samples, and we normalized by the number of CpGs measured in that gene. This gave us a standardized measure of conservation, *C_g_*, for the *g*th gene that controls for the number of CpG sites, *n_g_*, measured in that gene.

More formally, suppose that we have *S* samples for which we have measured methylation values at *n_g_* CpG sites for a given gene and that we denote the data obtained by the *S* × *n_g_* matrix *D*, where the (i,j)th element of the matrix, denoted by *d_ij_*, records the methylation measured for the *i*th sample at the *j*th CpG site. Then, the Manhattan (or pairwise) distance between samples *i* and *j* is defined as $M_{ij}=\sum_{k=1}^{n_{g}}\left|d_{ik}-d_{jk}\right|.$We then define the overall Manhattan distance for the set of samples at these sites (ie, for gene *g*) to be the average of those values—that is, $M_{g}=\sum_{i<j}^{}M_{ij}/H,$ where *H* = *S*(*S* − 1) / 2 is the number of distinct pairs of samples that can be formed. To control for the number of CpGs in that gene, we then normalize this value and work with *C_g_* = *M_g_* / *n_g_* moving forward.

Our next task is to determine which of those genes are the most conserved (ie, have the lowest values of *C_g_*). One approach would be to simply rank the calculated *C_g_* values and select the *L* smallest, for some choice of *L* (these are the most conserved in a “per CpG” sense). However, under the null hypothesis, $H_{0}$, that there is no difference in conservation across genes, the variance of the observed *C_g_* value for gene *g* will be inversely proportional to *n_g_*, the number of CpGs measured in that gene. As such, we expect an over-representation of genes for which the number of measured CpGs is low. This is what we observed in practice. Thus, and given that the *n_g_* values do vary greatly for the MethylationEPIC BeadChip microarray, our tool instead offers two bootstrapping^9^ approaches for this task, which we now describe.

For each observed value of *n_g_*, we constructed a null distribution for the measured value *C_g_* using a boot-strapping procedure. Specifically, we began by constructing a subset of all measured CpGs that includes only those CpGs that were annotated as gene-linked per the EPIC microarray documentation. We then repeated sample sets of *n_g_* CpG sites to act as “null genes” in the following ways:

In the approach we call the “naive bootstrap,” we proceeded as follows, repeating these steps for *l* = 1,…,*N_s_*, for some large number *N_s_*:

1. Sample a set of *n_g_* of these gene-associated CpGs independently at random, without regard to location, to form each null gene, *g_l_*.
2. Calculate the normalized Manhattan distance for null gene *g_l_*, as described in Statistical Approach. Denote this value by ${\hat{C}}_{l}$.

In reality, it is typically the case that the methylation status in neighboring CpGs is correlated (ie, neighboring CpGs are more likely to have the same methylation state than non-neighboring CpGs). Null genes constructed in the manner described by the naive bootstrap approach will not respect this correlation structure present in CpG sites actual genomes and, as such, may perform badly when CpG information is available densely across the genome, as in our data (we illustrate this in the Results). For that reason, we offer a second, more nuanced approach, which we refer to as the “adjusted bootstrap.” In this version of the bootstrap, we proceeded as follows:

First, we extracted all possible sets of *n_g_* consecutive CpGs, such that all *n_g_* CpGs are associated with the same gene. We did this for every gene and denoted this total set across all genes by $S_{n_{g}}.$ Again, we next repeated the following steps for *l* = 1,…,*N_s_*, for some large number, *N_s_*.

1. Sample a set of *n_g_* consecutive CpGs from $S_{n_{g}}.$ Denote this “null gene” by *g_l_*. The CpGs sites it contains will, by construction, all be associated with the same gene and will maintain the correlation structure that is typical among nearby CpG sites.
2. Calculate the normalized Manhattan distance for null gene *g_l_*, as described in Statistical Approach. Denote this value by ${\hat{C}}_{l}$.

This adjusted bootstrap procedure tested the same null hypothesis as the naive bootstrap but used sets of CpGs that better reflected the correlation structure typically found within the genome. In the results we report here, we repeated these bootstrapping procedures *N_s_* = 1,000 times for each distinct value of *n_g_*. We note that the “null gene” sampled in step 2 typically will be different in each repetition of that step. We then took the set of *N_s_* values of ${\hat{C}}_{l}$we generated for each distinct value of *n_g_* and used those as the null distribution from which we assessed the significance level of the observed values *C_g_* for all genes for which the measured number of CpGs is *n_g_*. The significance level is defined as the quantile of the observed value *C_g_* in this null distribution. The lower the quantile, (which can be thought of as a *P* value), the more significantly conserved the gene is. We extracted all genes, of all lengths, that fell between the 0th and 5th quantile and took them through to the next stage of the analysis, in which we compared them to gene expression values (described in the comparison with public data).

To better understand the importance of genes that are called highly conserved using the procedure described in this section, we then applied a gene-set enrichment analysis using the ReactomePA software package.^10^ This flagged pathways that were significantly enriched among our set of conserved genes.

In addition to conducting this analysis for each gene in its entirety (ie, including all CpGs that are associated with that gene), we also conducted analyses in which we considered particular genic regions (5′ untranslated region, or 500 and 2,000 base pairs [bps] from the transcription start site) to see whether these more localized regions might better correlate with gene expression. Finally, we also compared gene expression with conservation in the promotor region.

#### Comparison with public data.

The ultimate step of this analysis is to assess how well gene conservation correlates with gene expression. Because we had no expression data for the samples we used, we instead used data from Expression Atlas.^10a^ From there, we obtained expression levels for each gene in normal tissue for each of the colon, small intestine, and endometrium, calculated from RNA-seq data for tissue samples of 122 human individuals.^11^ Although the samples are different, the tissue is the same, which led us to believe that, for most genes at least, expression would be similar in their and our samples. Clearly, any correlation between conservation and expression that we would see in our actual data (were expression data available for our samples) would likely be higher than when comparing it with expression in unrelated public samples. As such, our test for correlation is likely to be conservative, but it is for this reason that we focused this proof-of-principle analysis on normal tissue rather than on tumor tissue. Ultimately, we do hope to apply this approach to tumor tissue as well.

## RESULTS

Every cell has its own epigenome. By comparing epigenomes between cells within an individual or between individuals, we can discover if certain regions are more conserved. As a first step, we illustrated that conservation is nonuniform along the genome, with greater conservation within genes (Table 1). In the table, we showed the mean value of the per-CpG Manhattan distance, *C_g_*, as a function of category. We categorized the sites in several ways: (1) as gene/nongene; (2) according to whether they fell in CpG islands, shores, shelves, or sea^12^; (3) whether they were located in the 5′ untranslated region of a gene; and (4) whether they were located within 1,500 bps or 200 bps of the transcription start site. As expected if conservation is a beacon or indicator of biologic function, conservation was significantly greater (ie, values of *C_g_* were low) inside genes versus outside of genes, with the greatest conservation observed within 200 bps of the transcription start site (Table 1). These conservation patterns were present for all three human tissues. Furthermore, we note that there was a strong correlation between conservation and genomic annotation of the region as CpG island/non-island. CpG islands are regions that are observed to have low levels of methylation. As such, it is not entirely surprising that methylation conservation should be high. Regions nearby to islands are often annotated as “shore” (closest to island) and “shelf” (between shore and sea). From the table, though, we see no evidence for increased conservation in these regions.

**TABLE 1.**
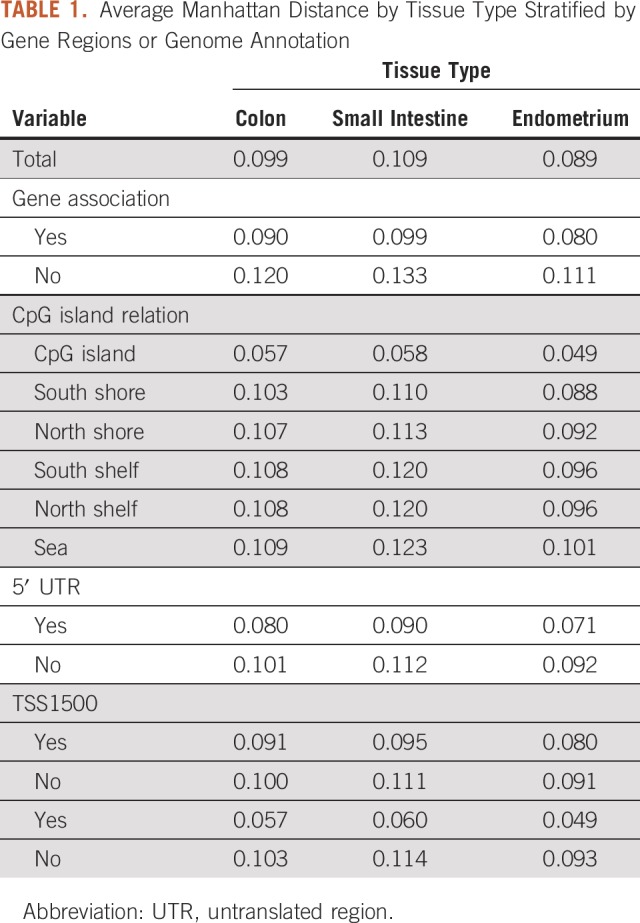
Average Manhattan Distance by Tissue Type Stratified by Gene Regions or Genome Annotation

**FIG 1.**
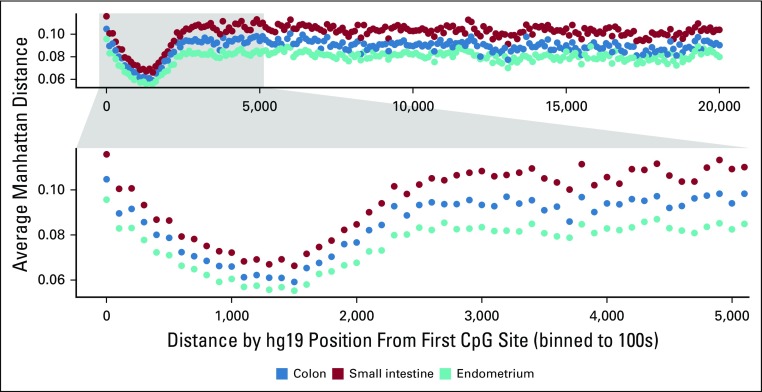
Average Manhattan distance for single CpGs as a function of position relative to first 5′ annotated gene CpG site. The greater conservation (lower average Manhattan distances) around genes indicates DNA methylation conservation generally extends for hundreds of base pairs and is not isolated to a single CpG site.

In Figure 1, we showed the behavior of conservation of CpGs sites as a function of their position relative to their associated gene but averaged across all genes. Each point shows the mean of the absolute value of the difference in methylation frequencies at a given CpG site across all samples. We grouped CpG sites according to their physical position, where 0 represents the location of the first CpG associated with a given gene (per the annotation file for the EPIC array) value. We see that the Manhattan distance is minimized (and, therefore, conservation is maximized) within the first 2,000 bps or so before then increasing to a steady value along the rest of the gene. The pattern is replicated across three tissue types, albeit at a slightly different level for each type.

**FIG 2.**
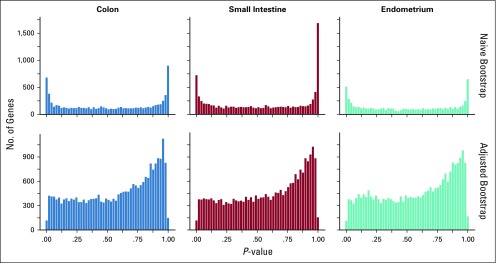
Distribution of boot-strapping *P* values for genes. Each column corresponds to a specific tissue. The top row shows results from the naive bootstrap procedure, whereas the bottom row shows the adjusted bootstrap results.

In Figure 2, we showed how the two methods of bootstrapping described earlier gave different *P* value distributions when applied to our data. The first row is the distribution one gets when CpG sites are randomly picked when constructing the null distribution of similar genes (the “naive” method). This setup leads to the vast majority of genes having an observed *C_g_* value that either is always bigger or is always smaller than the “null” genes created by the bootstrapping procedure. Because we wished to rank genes according to *P* value, this setup was problematic, because it essentially resulted in a large number of ties along with an over-representation of *P* values equal to 0 or 1. The second row shows the distribution of the *P* values resulting from the “adjusted bootstrap” procedure. This distribution is much more uniform, as desired, and has far fewer ties between *P* values, which enabled us to rank the genes more effectively. For this reason, we used the adjusted method to produce the results shown in the rest of this paper.

**FIG 3.**
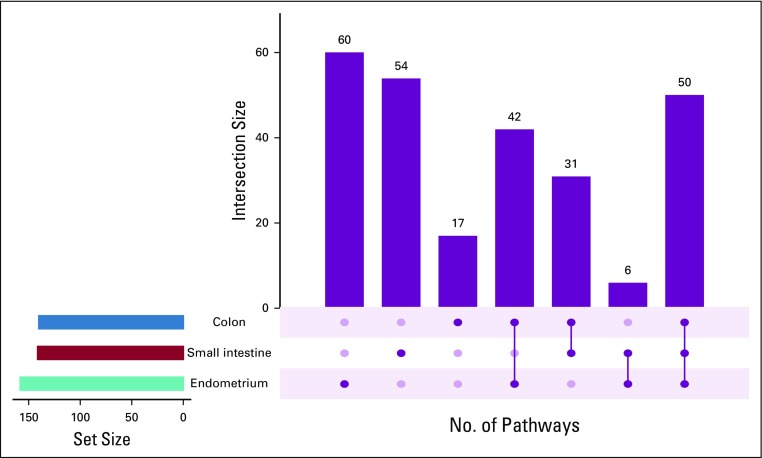
Results of pathway conversation analysis. The first three columns show the number of pathways that are called as significantly over-represented just in a single tissue type, while the next four columns show how many pathways that are called as conserved in two or more tissue types. The overall number of pathways called as significantly conserved in each tissue is shown by the colored bars at bottom left.

**FIG 4.**
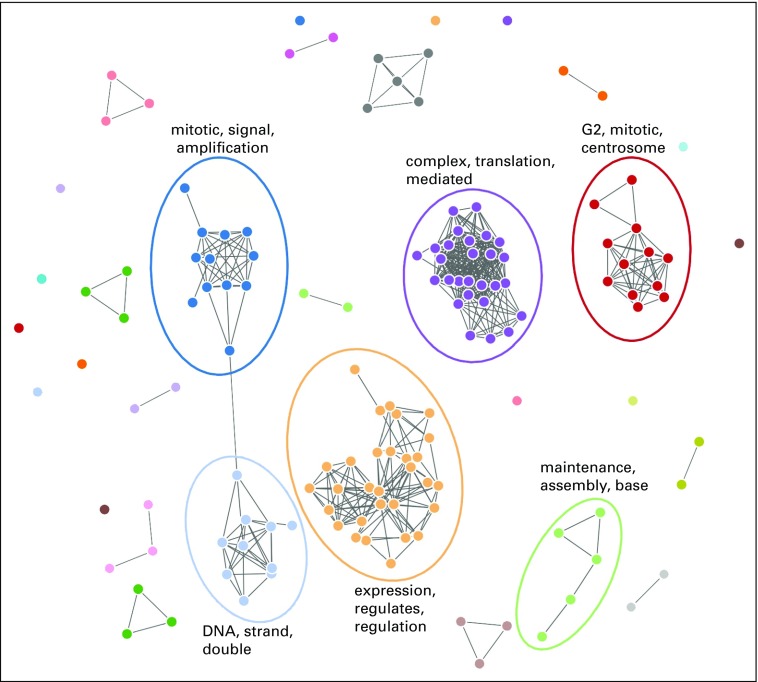
Enrichment map of pathways of conserved genes in small intestine tissue. Edges are shown between pathways if the overlap ratio is > 0.5. Major clusters are labeled according to most frequent words in pathway names.

Epigenetic gene conservation can be further stratified or ranked, because not all genes are expressed in all tissues. Therefore, conservation should vary between tissues. To explore this, after calculating the *C_g_* values, we took the 5% of genes that were most conserved for each tissue, separately for each tissue, and then conducted a gene-set enrichment analysis to see what pathways were over-represented among those genes. We referred to these pathways as “conserved pathways.” We then determined how many of the conserved pathways were conserved in one, two, or all three tissues. Figure 3 shows the results of this analysis. The endometrium and small intestine had the greatest numbers of uniquely conserved pathways, but the overlap between these pathways was small (just five pathways). However, interestingly, a core group of 50 pathways were conserved in all three tissues. These pathways are enriched in core housekeeping functions (cell cycle, DNA replication, transcription, translation) that are essential to all mitotic cells (Fig 4). This reinforces the idea that we are successfully using conservation of methylation to detect genes, and then pathways, that play important roles in the tissue concerned.

The enriched gene-sets can be organized into a network. In Figure 4, we give an example using the most over-represented pathways in the small intestine. In the figure, the nodes represent pathways that were labeled as conserved in our analysis. The edges between nodes represent wether genes are associated with both pathways that are labeled as highly conserved in our analysis. If the overlap proportion of genes between pathways is less than 0.5, no edge is present.^13^ Again, we see that most of the pathways that we detected as most conserved have significant overlaps in genes involved, likely because they perform key housekeeping roles in cell function.

**FIG 5.**
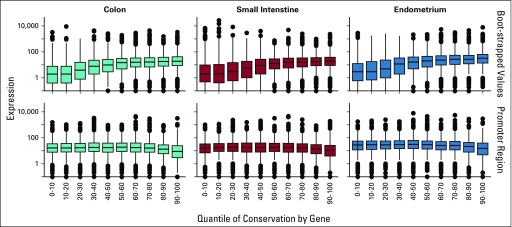
The relationship between conservation and expression. Genes are collected into 10 groups according to the degree of conservation measured in our data. For each group, we then show a box-plot of the distribution of log (gene expression) values recorded for the corresponding tissue type in the Expression Atlas database. Columns correspond to the tissue type. The top row shows results when assessing conservation for the entire gene; the bottom row shows the results when assessing conversation just for the promoter region of each gene. We see that gene conservation correlates with expression better than does promoter conversation.

Finally, we examined whether conservation correlates with gene expression, our proxy measure of importance of a gene. Although variation in gene promoter methylation often is associated with gene silencing, the degree to which it correlates with gene expression is unclear.^14^ As seen in Figure 5, for all three tissues, we found that conservation did correlate with gene expression levels and performed better as an indicator of expression than did gene promoter methylation, which did not appear to correlate with expression at all. The first row represents the adjusted conservation values obtained with our adjusted bootstrapping approach (*x*-axis, binned by value), whereas the second row is the mean methylation in the promoter region (*x*-axis, binned by value). The expression values (*y*-axis) were taken from the Expression Atlas^6^ and are displayed on a logarithmic scale. The values for conservation and mean methylation in promoter were binned in such a way that an equal number of points were placed in each bin. Higher bin number represents more conserved genes (ie, conservation increases as we move from left to right in the figure).

## DISCUSSION

The Methcon5 R package that we introduced here^15^ provides software necessary to carry out the calculations for conservation and the bootstrapping procedure. The functions have been split into two sections: (1) calculations of the conservation value by region and (2) bootstrapping methods to calculate *P* values on the basis of conservation values and region length. The calculation of the conservation value is customizable, with user-provided functions allowed and with a default for arithmetic mean. Currently, three different bootstrapping methods are included in the package, two of which have been described in this paper. Also, a second repository is available, which includes all of the analysis scripts necessary to reproduce the analysis performed here, starting from IDAT files.^16^ The analyses in this paper took < 30 minutes to run on a Macbook Pro.

A priori, it has been found that conserved genomic regions tend to be functional.^3^ Using the software we introduced here, we applied this principle to epigenomes and presented novel software to identify and rank CpG DNA methylation conservation along the human genome. Conserved genomic regions likely reflect selection, and therefore the identification of preferentially conserved epigenetic regions potentially can identify the genes that are most important to the function of a cell—a frequent goal of biologic investigations.

The example analysis presented here illustrates that known functional genomic regions have greater epigenetic conservation. In principle, such epigenetic conservation can be used to help identify which genes are more critical to the survival of a cell. Interestingly, function appeared to correlate with conservation of methylation at multiple gene-associated CpG sites (Fig 1), which may indicate that the epigenetic configuration of the gene region and not of a specific CpG site is informative. The approach and software require at least two samples from the same population. Better still, the comparisons can be applied to a range of samples. Potentially, epigenetic conservation can indicate what genes are under greater selection in native human tissues. For example, comparisons of epigenomes between opposite sides of the same human colorectal cancer reveal preferential conservation of genes involved in immune surveillance, suggesting that it is critically important for cancers to hide from the immune system.^17^ Although conservation per se does not conclusively indicate function, highly conserved epigenetic regions can serve as beacons for unbiased discovery of genes or noncoding regions that are more likely to be critical to the function or survival of human cells. The data used in this analysis are available upon request.
